# Effects of nicotinamide riboside supplementation during late gestation and lactation on sow performance, milk metabolome, and gut microbiome

**DOI:** 10.1186/s40104-025-01339-x

**Published:** 2026-02-10

**Authors:** Long Huang, Xiaohan Yang, Chenglin Pan, Wei Zhang, Yingjie Li, Ruilan Zhang, Hua Li, Ying Li, Yong Zhuo, Xuemei Jiang, Lianqiang Che, Yan Lin, Shengyu Xu, Zhengfeng Fang, Bin Feng, De Wu, Lun Hua

**Affiliations:** 1 Key Laboratory of Animal Disease-Resistant Nutrition of the Ministry of Education of China, Key Laboratory of Animal Disease-Resistant Nutrition of Sichuan Province, Animal Nutrition Institute, Sichuan Agricultural University, 211 Huimin Road, Wenjiang District, Chengdu, 611130 PR China; 2 Guangdong Provincial Key Laboratory of Animal Molecular Design and Precise Breeding, College of Life Science and Engineering, Foshan University, Foshan, 528000 China

**Keywords:** Microbiota, Milk, NAD^+^, Nicotinamide riboside, Reproductive, Sow

## Abstract

**Background:**

Nicotinamide riboside (NR) supplementation has been demonstrated efficacy in enhancing female reproductive outcomes, but its regulatory role in sow performance and gut microbiome remains undefined. This study systematically evaluated the impacts of dietary NR supplementation during late gestation and lactation on sow performance and gut microbiome remodeling. A total of 280 sows were randomized assigned to one of four groups: a control group fed basal diet or one of three groups receiving NR-supplemented diets (2, 4, or 8 g/d; *n* = 70/group). Sow reproductive performance, blood metabolic parameters, milk metabolome, and fecal 16S rRNA sequencing were measured.

**Results:**

Maternal NR supplementation linearly shortened farrowing duration (*P* < 0.01) and tended to decrease the incidence of intrauterine growth restriction and the number of late gestation mummies (*P* < 0.1), while concurrently increasing the within-litter uniformity (*P* = 0.1). Litter weaning weight and average daily gain increased quadratically with NR dosage (*P* < 0.05). NR supplementation orchestrated plasma metabolite regulation (triglycerides and total cholesterol; *P* < 0.05), enhanced antioxidant biomarkers (T-AOC, GSH-Px, T-SOD; *P* < 0.05), and reduced inflammatory cytokines (TNF-α; *P* < 0.05) across gestation and lactation. Milk yield, colostrum/milk dry matter, crude protein, and crude fat were increased (*P* < 0.05), together with higher levels of NAD^+^ metabolites (NAD⁺, NR, nicotinamide) and beneficial bioactive factors (milk polar lipids, 3-aminosalicylic acid, fenugreekine; *P* < 0.05). Gut microbiota analyses at lactation day 14 revealed NR-enriched beneficial taxa (*Bifidobacterium*, *Ruminococcus*, *Lachnospiraceae*, *Subdoligranulum*, *Clostridium butyricum*, *Succiniclasticum*) across sow-offspring dyads, which was associated with the activation of microbial NAD⁺ enzymes (*NadR*/*NAMPT*; *P* < 0.05) and enhancement of systemic short-chain fatty acid flux, notably an increase in plasma butyrate acid (*P* < 0.05).

**Conclusion:**

Maternal supplementation of NR during late gestation and lactation increases sow performance and promotes gut NAD^+^ metabolic-associated microbiome remodeling. These findings propose maternal NR intervention as a novel strategy to enhance mammary lactogenesis and lactation metabolism in swine production, with potential applications for therapeutic strategies for lactation insufficiency.

**Graphical Abstract:**

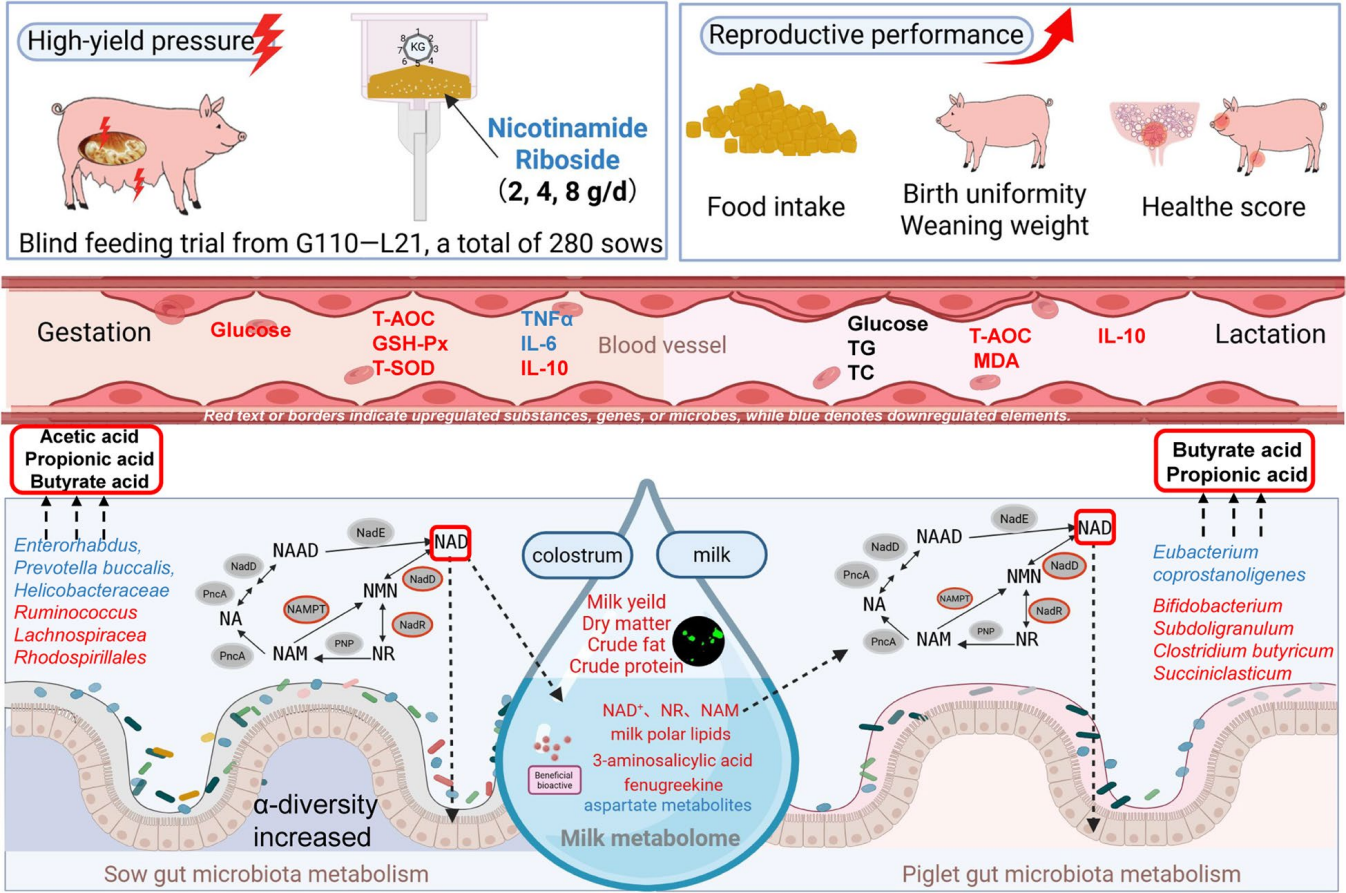

## Introduction

Hyper-prolific sows face multifaceted late-gestation stressors [1] that compromise pregnancy outcomes and lactation performance [2, 3]. Uterine expansion, coupled with accelerated fetal development, heightens metabolic/oxygen demands, which in turn elevates systemic inflammation and redox imbalance [4, 5], and promotes placental mitochondrial dysfunction, ultimately impairing birth outcomes [6–8]. Subsequently, such gestational dysregulation also undermines mammary secretory function—lactation creates a high metabolic demand in the mammary gland, necessitating numerous mitochondria as redox-active hubs to support the biosynthesis of milk components [9, 10]. Both our data and previous studies have linked mitochondrial damage to oxidative stress and to reductions in fetal growth and lactational capacity [7, 8, 11]. Consequently, nutritional interventions spanning late gestation through lactation are essential to mitigate metabolic imbalances in both circulation and organ cellular environments.

Nicotinamide adenine dinucleotide (NAD⁺) is an essential cellular metabolite derived from vitamin B_3_ forms and functions as a redox cofactor and a central regulator of mitochondrial homeostasis [12]. NAD⁺-targeted therapies have shown promise against obesity, inflammation and intestinal dysfunction in clinical settings [12–14], but their application in livestock with complex metabolic demands remains underexplored. Conventional vitamin B_3_ supplements (niacin or nicotinamide) give inconsistent results, likely due to variable efficiency in converting precursors to NAD⁺ [15–17]. By contrast, nicotinamide riboside (NR), an NAD⁺ precursor could utilize the salvage pathway rather than the Preiss-Handler pathway employed by niacin (NA) or nicotinamide (NAM), bypassing rate-limiting enzymatic steps and improving conversion efficiency [12, 18]. NR administered both orally and intravenously significantly elevate circulating and tissue NAD⁺ levels, achieving superior systemic bioavailability [19].

Emerging evidence indicates heightened maternal NAD⁺ demand during gestation and lactation [20, 21], suggesting NAD⁺ repletion safeguards reproductive function by preserving NAD⁺ pool equilibrium, mitochondrial homeostasis, and signaling cascades [21–23]. For example, NR reduced ovarian fibrosis and restored ovarian NAD⁺ and mitochondrial function in PCOS models [24], and oral NR improved fetal/placental growth and placental mitochondrial function while reducing inflammation and oxidative stress during pregnancy [25, 26]. These findings are consistent with our prior work showing NR-enhanced mammary development via *SIRT1*-mediated mitochondrial mechanisms [21]. Moreover, gut microbiota also critically modulates mammalian reproductive physiology [27]. An estimated substantial fraction (60%) of gut microbes participate in NAD⁺ metabolism, and studies have demonstrated that germ-free mice fail to elevate systemic NAD⁺ levels upon exogenous NAD precursor supplementation [19, 28], while microbial communities mediate host NAD⁺ bioavailability and also orchestrate bidirectional interplay related to microbiota-dependent metabolites (short-chain fatty acids, bile acids) that directly influence host immunity and energy metabolism [29]. Hence, we hypothesize that maternal NR supplementation may enhance reproductive performance by modulating gut microbial metabolic activity, thereby improving systemic host metabolism and positively influencing gestation and lactation outcomes. Accordingly, this study evaluated the efficacy of dietary NR supplementation in a large cohort to improve sow reproductive performance, concurrently assessing impacts on maternal metabolic homeostasis, milk metabolome, and gut microbiota-mediated metabolism to bridge nutrient strategies with perinatal optimization.

## Methods

Animal procedures were approved by the Animal Care and Use Committee of the Animal Nutrition Institute, Sichuan Agricultural University and complied with the current laws relating to animal protection (Ethics Approval Code: No. YYS20240825). Throughout the study, the sow and piglets were maintained under species-appropriate housing conditions. Sows received two deep intrauterine inseminations with semen from Duroc boars, ensuring pre-insemination sperm motility of no less than 80%, and were vaccinated following a standard protocol, including swine fever, parvovirus, and pseudorabies vaccines, adjusted based on local epidemics. Animal handling was performed by trained personnel to minimize disturbances, with food rewards and gentle handling provided during sampling procedures to reduce stress.

### Animals and experimental design

A total of 280 mixed-parity sows (parity 4.8 ± 1.8; Landrace × Yorkshire crossbreds) were enrolled at gestation day 90 (G90) and randomized, after matching by parity, breed, backfat (P2), body condition score (BCS), and effective nipples, into four treatment groups (*n* = 70/group): control (0 g/d NR), or NR group receiving 2, 4, or 8 g/d NR. The basal diet (mixed-grain) met or exceeded the National Research Council (NRC; 2012) [30] recommended nutrient requirements of sows (Table 1) [31]. This trial was conducted in a double-blind manner. Premixed powders with indistinguishable appearance but varying NR content (0%, 20%, 40%, and 80%) were prepared in advance. From G90 to lactation day 22 (L22), at both 0800 and 1500 h, 5 g of the respective powder was accurately weighed and manually top-dressed or mixed into individual sow feeders to achieve total daily NR intakes of 0, 2, 4, or 8 g. Gestation feed was 2.5–2.8 kg/d to maintain BCS 3.0–3.5; after farrowing lactation feed increased from 2.0 kg/d to ad libitum. Sows were individually housed (2.2 m × 0.6 m) during pregnancy, and then transferred to adjustable farrowing cages (2.4 m × 1.5 m) at G108 with cloprostenol-induced synchronized parturition management. Continuous farrowing supervision included intervention for > 45-min inter-delivery intervals or absent contractions. Litters were standardized within 24 h post-farrowing within treatments. All sows/piglets were given free access to water, and sow milk was the sole nutrient source for piglets. Biological samples were collected from 15 randomly selected sows per group (representative of cohort means for backfat/BCS). Dystocia cases were therapeutically managed and retained for performance data, but excluded from bio-sampling.

**Table 1:** Ingredients and nutrient composition of the basal diets for sows

Item	Gestation	Lactation
Ingredients, %		
Corn	20.00	47.01
Sorghum	35.03	19.60
Soybean meal	-	8.00
Sunflower meal	6.97	5.84
Copra meal	9.00	8.00
Wheat bran	15.70	-
DDGS	5.00	-
Beet pulp	3.00	-
Premix and Others^a^	5.30	11.55
Analysis nutrient composition^b^		
DM, %	86.47	86.94
Crude protein, %	13.73	17.55
Gross energy, Mcal/kg	3.73	3.90
Ether extract, %	3.22	5.52
Crude fiber, %	5.67	2.85
Neutral detergent fiber, %	17.55	12.08
Acid detergent fiber, %	10.30	7.09
Ash, %	7.24	6.30

^a^Premix and others ingredients consisted of dietary crystalline amino acids, minerals and vitamins that meet NRC (2012) [30] nutritional requirements for sows. The proprietary premix formulations were excluded from compositional disclosure due to intellectual property restrictions

^b^The data in the table derived from triplicate analytical determinations. The chemical composition of experiment diet was analyzed according to lab method

### Measurements of reproductive performance

Sow backfat depth (P2 measurement) was ultrasonically determined at 65 mm left of the dorsal midline (last rib level) using an ultrasonic device (Renco Lean-Meatier; Renco Corporation, Minneapolis, MN, USA). The numbers of piglets born alive, stillborn, and mummified, and their weights at birth, 24 h post-fostering, and lactation day 22 were recorded, as well as daily piglet mortalities. Total pigs born per litter were calculated as the sum of pigs born alive, stillborn, and mummified. IUGR was defined as birth weight < 1.5 SD of the litter mean, while LBW (low BW) newborn were defined as weighing less than 1.1 kg [32], with additional percentile analysis (10^th^ percentile) to contextualize weight distribution. Mummification timing was estimated using crown-rump length (CRL) according to the regression model of Wang et al. (CRL: 21.63 cm at G60 vs. 31.69 cm at G90) [33]. At delivery, farrowing kinetics were analyzed through total parturition duration (first-to-last neonate expulsion) and birth interval [farrowing duration/total born (except mummified)]. Moreover, sow rectal temperatures were serially measured at 48 and 72 h post-farrowing.

### Sow and piglet health score

Sow-piglet health assessments (mammary status, piglet skin lesions, diarrhea) [34] were conducted per litter on lactation days 7, 14, and 21. Clinical mammary examination involved udder inspection and palpation to evaluate regression (0 = in lactation; 1 = poorly formed/in regression; 2 = not formed/without milk production) and redness (0 = physiological skin color; 1 = moderate; 2 = intense), with grade 3 manifestations (typical of postpartum dysgalactia syndrome) rarely observed. Skin lesions were assessed using consistent criteria: knee lesions (> 0.5 cm diameter at carpal joints) were scored 0 (none), 1 (< 50% piglets affected), or 2 (≥ 50%); facial scabbed wounds (> 2 cm diameter on forehead-nasal bridge) followed identical scoring; smaller lesions were excluded. Piglet diarrhea was scored as: 0 = solid/well-formed, 1 = soft/formed, 2 = fluid/yellowish, 3 = watery/projectile.

### Sample collection

Sow blood was collected via the ear vein on gestation day 110 (G110) and lactation day 14 (L14), with piglet jugular vein blood sampled from mean-weight-matched littermates. All blood samples were centrifuged (3,500 × *g*, 15 min, 4 °C), with plasma aliquots stored at −20 °C for analysis. Fecal samples (2 g) were aseptically collected from sows and piglets on L14 via rectal massage and rectal grab method. The entire procedure was performed under sterile conditions, including the use of sterilized gloves and pre-sterilized cryovials. All samples were immediately placed in liquid nitrogen for temporary storage after collection. Piglet samples were obtained from sex-balanced cohorts to minimize the potential influence of sex. Colostrum was manually collected 2 h after the first piglet’s birth, while milk was obtained on L14 after oxytocin administration. Mammary milk from anterior/middle/posterior glands was pooled. Fecal/milk samples were snap-frozen (−80 °C), and milk subsamples were stored at −20 °C for nutrient composition analysis.

### Blood biochemical, antioxidant and cytokine assays

Plasma metabolic biomarkers in sows, including glucose (GLU), non-esterified fatty acids (NEFA), triglycerides (TG), total cholesterol (TC), urea, alkaline phosphatase (ALP), alanine aminotransferase (ALT), and aspartate aminotransferase (AST), were quantified using an automatic biochemical analyzer (Hitachi 7020, Tokyo, Japan). Oxidative stress parameters were systematically assessed by measuring plasma catalase (CAT) activity, superoxide dismutase (SOD), glutathione peroxidase (GSH-Px), total antioxidant capacity (T-AOC), and the lipid peroxidation marker malondialdehyde (MDA) using standardized assay kits (Nanjing Jiancheng Bioengineering Institute, China). The concentrations of tumor necrosis factor-α (TNF-α), interleukin-10 (IL-10), and interleukin-6 (IL-6) were determined using enzyme-linked immunosorbent assay (ELISA) kits from the same manufacturer.

### Short-chain fatty acid (SCFA) determination

Plasma SCFA concentrations were quantified as previously described [35], using a Varian CP-3800 gas chromatography system (Varian Medical Systems, Palo Alto, CA, USA) configured with a flame ionization detector (FID) and a capillary column. Briefly, serum (400 µL) was deproteinized using 50 µL 25% (w/v) metaphosphoric acid plus 4 µL crotonic acid (210 mmol/L, as an internal standard), vortexed, incubated at 4 °C for 30 min, and centrifuged at 15,300 × *g*, for 10 min. 100 µL of supernatant was mixed with 100 µL methanol, centrifuged (42,500 × *g*, 15 min), and filtered through a 0.22-µm membrane filter prior to GC analysis.

### Milk composition analysis and lipid droplet staining

Colostrum and milk composition (dry matter, crude protein, true protein, crude fat, lactose, urea nitrogen) were quantified using a MilkoScan FT2 analyzer (Foss, Hillerød, Denmark) on samples stored at −20 °C. Milk lipid droplets (stored at −80 °C) were stained with 0.1% BODIPY 493/503 (1:100 v/v; Thermo Fisher), incubated for 30 min in the dark (25 °C), and imaged via fluorescence microscopy (Olympus DMI400B, Japan). Analyses included mean droplet area and proportion of total lipid droplet area. Piglet daily weight gain (ADG) was estimated from milk composition using the regression model of Hojgaard et al. [36]: piglet weight gain d 3–25 (g/d) = −70.2 + 14.1 × milk protein (%) + 0.24 × milk intake (g/d) or 2.60 + 1.93 × milk protein intake (g/d) + 2.75 × milk lactose intake (g/d).

### Milk metabolome

Milk samples (100 µL) were mixed with methanol containing deuterated internal standards (1:1), vortexed (30 s), sonicated (4 °C, 10 min), incubated (−40 °C, 1 h), and centrifuged (13,800 × *g*). Supernatants were subjected to LC–MS/MS analysis (Vanquish UHPLC; Thermo) with a Waters BEH Amide column (2.1 mm × 50 mm, 1.7 μm) coupled to an Orbitrap Exploris 120 mass spectrometer (IDA mode; Xcalibur-controlled). Raw data were converted to mzXML format via ProteoWizard and processed with an XCMS-based R script for peak detection, extraction, alignment, and integration, with metabolite identification against BiotreeDB v3.0. Metabolomic data were log-transformed and standardized to reduce noise and variable heterogeneity. Pathway analysis was performed using KEGG, MetaboAnalyst, and OmicStudio. False discovery rate was controlled using the Benjamini–Hochberg method (FDR < 0.05). PCA was performed using FactoMineR/ggplot2 in OmicStudio.

### Fecal microbial analyses

Genomic DNA from sow/piglet feces was extracted using the E.Z.N.A.^®^ Soil DNA Kit (Omega Bio-tek) and subjected to quality control. The V3–V4 region of bacterial 16S rRNA was amplified (primers 338F/806R; BIO-RAD T100 Thermal Cycler), purified (PCR Clean-Up Kit; Shandong YuHua Biomedica Institute, China), and quantified (Qubit 4.0). Sequences were quality-filtered with fastp (v0.19.6) and merged with FLASH (v1.2.11), followed by DADA2 denoising in QIIME2 (v2020.2) to generate amplicon sequence variants (ASVs). Taxonomy was assigned using QIIME2’s Naive Bayes classifier with SILVA database (v138). Metagenomic functions were predicted via PICRUSt2 (Phylogenetic Investigation of Communities by Reconstruction of Unobserved States) [37] based on ASV sequences. Statistical analyses utilized Majorbio Cloud (www.majorbio.com), with LEfSe identifying significantly enriched taxa (LDA > 2, *P* < 0.05; phylum-species).

### Statistical analysis

Data were analyzed using the MIXED and GLIMMIX procedures in SAS 9.4 (SAS, Cary, North Carolina, USA), with the sow as the experimental unit. Summary statistics were evaluated via PROC UNIVARIATE, defining outliers as observations beyond ± 3 SD. The initial model included breed as a random effect and diet, parity, and diet × parity as fixed effects. Based on the non-significant diet × parity interaction (*P* > 0.05), the interaction term was removed. The final model only kept diet treatment as fixed effects, with parity and breed as random effects. The model for the analysis was *Y*_*ij*_ = *μ* + *F*_*i*_ + *e*_*ij*_, where *Y*_*ij*_ is an observation of the dependent variable *ij*, *μ* is the population mean for the variable, *F*_*i*_ is the effect of NR supplement, as a fixed effect, and *e*_*ij*_ is the random error. Degrees of freedom were approximated using the Kenward-Roger method (*DDFM* = *KR*). Residual normality and homoscedasticity were verified via PROC UNIVARIATE with NORMAL and PLOT options. Variables violating assumptions (litter size, stillborn rate, pig mortality, health score) or following binomial/Poisson distributions were analyzed using PROC GLIMMIX. Orthogonal contrasts tested linear/quadratic effects of NR supplementation, with coefficients adjusted for unequal treatment spacing. Least square means using the LSMEANS statement were reported for all other variables, and multiple comparisons were made using adjusted Tukey method. Additionally, preplanned contrasts (C vs. NR) were used to compare pooled NR supplementation groups (encompassing all dosage gradients) with controls. All results were considered significant at *P* < 0.05 and considered a tendency at *P* ≤ 0.10. Sows excluded from analyses due to leg lesions, febrile illness, or abortion were proportionally balanced across groups, and the number of observations per dietary treatment ranged from 65 to 70.

## Results

### The cohort characteristics and growth performance of sows

As shown in Table 2, there were no significant differences on G90 estimated BW [38] and backfat depth, but NR supplementation demonstrated dose-dependent effects on backfat depth change between G90 and G110, where sows that received 4 g/d NR exhibited minimized backfat thickness loss (quadratic, *P* < 0.05). When compared to controls, sows fed NR had higher ADFI at weeks 2–3 and TFI during lactation with 4 g/d NR exhibiting the highest food intake (quadratic, *P* < 0.05), but sows received 8 g/d NR had the lowest ADFI at the first week.

**Table 2:** The growth performance of late gestation and lactation sows supplemented with increasing levels of nicotinamide riboside

Items	NR supplement, g/d				SEM	*P*-values		
	0	2	4	8		Lin	Quad	C vs. NR^1^
Start sows, n	70	70	70	70				
End sows, n	65	63	67	68				
Average parity	3.76	3.85	3.76	3.71	0.32	0.681	0.437	0.578
BW at G90^2^, kg	263.0	266.0	265.7	265.1	7.09	0.715	0.538	0.455
G90 BF, mm	12.98	12.52	13.03	12.90	0.37	0.860	0.805	0.666
G110 BF, mm	12.73	12.56	12.99	12.91	0.29	0.555	0.916	0.831
L21 BF, mm	12.17	12.46	12.64	12.50	0.48	0.510	0.428	0.344
BF change, mm								
G90–G110	−0.23^a^	−0.07^ab^	−0.01^b^	−0.13^ab^	0.09	0.366	0.022	0.032
G110–L21	−0.48	−0.23	−0.38	−0.42	0.22	0.906	0.323	0.349
ADFI 1 week, kg	5.88^b^	5.93^b^	6.72^a^	5.25^c^	0.17	0.011	< 0.01	0.674
ADFI 2–3 weeks, kg	8.94^bc^	9.46^ab^	9.76^a^	8.82^c^	0.14	0.281	< 0.01	0.014
TFI, kg	157.91^c^	165.19^b^	173.42^a^	152.49^c^	7.76	0.079	< 0.01	0.017

*Lin* Linear effects, *Quad* Quadratic effects, *BCS* Body condition, *BF* Backfat depth, *BW* Body weight, *G90* Gestation day 90, *G110* Gestation day 110, *L21* Lactation day 21, *ADFI* Average daily food intake, *TFI* Total food intake

^1^C vs. NR means that the comparative analysis of pooled NR supplementation groups (encompassing all dosage gradients) compared with controls

^2^The sow BW was predicted using the best fit multiple regression models, BW = −133 + 3.77 parity + 0.32 day + 1.72 BF + 0.23 heart girth. Heart girth (mm) was defined as the circumference of the sow immediately behind the front legs and in front of the first mammary glands

^a,b,c^Means within a row with different superscripts differ using adjusted Tukey method (*P* < 0.05)

### Maternal NR supplementation improved litter uniformity and reduced the late gestation mummies and farrowing duration

As presented in Table 3, no significant differences were observed in total born, live-born, stillborn rate, or mummified piglets between groups. However, sows fed NR linearly tended to reduce mummification in late gestation (*P* = 0.057). Meanwhile, NR administration trended toward reducing the litter CV_BW_, IUGR, and LBW born (< 1.1 kg; *P* < 0.1), with LBW born reduced linearly in response to NR dosage (*P* = 0.036). Although total litter weight and individual birth weight did not differ across treatments, NR supplementation significantly increased the 10^th^ percentile birth weight (+ 8.73% vs. controls; *P* = 0.046). The cohort demonstrated an average farrowing duration of 185.98 ± 7.35 min, with maternal NR supplementation linearly shortening parturition time (*P* = 0.006) and birth interval (*P* = 0.056), achieving the shortest farrowing duration at 8 g/d NR, numerically. Postpartum assessments indicated no pyrexia (rectal temperature > 39.5 °C) at 48 or 72 h post-farrowing, and NR treatment exerted a dose-dependent reduction in body temperature at 48 h postpartum (*P* = 0.040).

**Table 3:** The litter performance of late gestation and lactation sows supplemented with increasing levels of nicotinamide riboside

Items	NR supplement, g/d				SEM	*P*-values		
	0	2	4	8		Lin	Quad	C vs. NR
Total born, n	16.10	15.98	15.86	15.98	0.40	0.848	0.723	0.743
Born alive, n	14.33	14.31	14.22	13.97	0.35	0.364	0.826	0.642
Stillborn rate^1^, n	7.94	8.32	7.91	9.02	1.33	0.533	0.776	0.720
Mummified, n	0.23	0.22	0.23	0.21	0.08	0.913	0.915	0.949
LG mummies^2^, n	0.14^a^	0.05^ab^	0.04^b^	0.02^b^	0.04	0.057	0.238	0.028
IUGR, n	1.38	1.09	1.28	1.00	0.11	0.105	0.974	0.094
LBW born, n	5.37^b^	4.72^a^	5.10^a^	4.40^a^	0.06	0.036	0.909	0.051
Litter CV_BW_^3^, %	24.02^a^	22.24^ab^	23.13^ab^	21.88^b^	0.74	0.146	0.740	0.100
Newborn weight, kg								
Live at birth	1.36	1.40	1.37	1.41	0.03	0.292	0.926	0.258
Litter 10^th^ percentile^4^	0.821	0.901	0.881	0.896	0.03	0.177	0.266	0.046
Litter of born alive	19.37	19.90	19.15	19.55	0.49	0.975	0.932	0.773
Farrow duration, min	210.30^a^	179.98^b^	180.80^b^	172.79^b^	8.59	0.006	0.111	0.001
Birth interval, min	13.55^a^	11.65^b^	11.61^b^	11.37^b^	0.68	0.056	0.153	0.013
Farrowing RT 48 h, °C	38.92^a^	38.43^b^	38.70^ab^	38.86^ab^	0.10	0.040	0.451	0.168
Farrowing RT 72 h, °C	38.45	38.47	38.58	38.39	0.06	0.336	0.110	0.159

*IUGR* Intrauterine growth restriction, *LBW* Low body weight, *LG* Late gestation, *CV*_*BW*_ Coefficient of variation of birth weight, *RT* Rectal temperature

^1^Number of stillborn piglets out of total born (sum of born alive, still born, and mummified)

^2^The mummy, measuring surpass 31 cm in head-rump length, is determined to have formed after gestation day 90

^3^ Litter CV_BW_ = (*σ/μ*) × 100. *σ* is the SD and *μ* is the average birth weight of litter

^4^10^th^ percentile body weight per litter

^a,b^Means within a row with different superscripts differ using adjusted Tukey method (*P* < 0.05)

### Maternal NR supplementation affected the weaning weight of piglets and milk production

As shown in Table 4, no significant differences were observed in the sow effective nipple, within-litter size, and weight after 24 h cross-fostering among groups. Compared with controls, NR supplementation linearly reduced the pre-weaning mortality (*P* = 0.025) and increased the number of weaned piglets (*P* = 0.021). Meanwhile, NR significantly increased weaning weight (+ 273 g vs. controls; *P* = 0.028) with peak response at 4 g/d (linear,* P* = 0.021 and quadratic,* P* = 0.073). Owing to combined effects on weaned piglet number and weight, NR-supplemented sows exhibited significantly increased litter weight (*P* = 0.014), litter ADG (*P* = 0.042), and milk production (*P* = 0.019) versus controls, where the greatest response was observed at 4 g/d (linear and quadratic,* P* < 0.05).

**Table 4:** The lactation performance of late gestation and lactation sows supplemented with increasing levels of nicotinamide riboside

Items	NR supplement, g/d				SEM	*P*-values		
	0	2	4	8		Lin	Quad	C vs. NR
After cross-foster^1^, n	12.44	12.46	12.74	12.57	0.20	0.289	0.206	0.262
Effective nipple, n	12.76	12.39	12.81	12.78	0.21	0.420	0.355	0.463
Pigs weaned^2^, n	11.33^b^	11.32^b^	11.90^a^	11.70^a^	0.27	0.021	0.134	0.063
Pig mortality, %	8.91	9.04	6.12	6.53	0.01	0.025	0.465	0.090
Piglet performance, kg								
Starting weight^3^	1.49	1.52	1.50	1.54	0.03	0.351	0.953	0.417
Weaning weight	6.31^c^	6.38^bc^	6.76^a^	6.61^ab^	0.13	0.020	0.073	0.028
Litter performance, kg								
Litter starting weight	18.87	19.37	19.46	19.71	0.72	0.206	0.654	0.214
Litter weaning weight	72.69^b^	72.87^b^	81.38^a^	78.11^a^	2.23	0.004	0.038	0.014
Litter ADG, kg	2.57^b^	2.55^b^	2.93^a^	2.75^a^	0.08	0.019	0.041	0.042
Milk production^4^, kg	215.64^b^	214.01^b^	248.22^a^	234.81^a^	6.13	0.004	0.039	0.019

*ADG* Average daily gain

^1^Litter size was standardized to 12–14 through cross-fostering of pigs within treatment within 24 h of parturition, and within-litter have similar weights to ensure that the competition for nipple

^2^On lactation day 22, litter size was counted and weighting

^3^Starting weight = litter weight/piglet number after cross-foster

^4^Estimated milk yield was calculated as 4 g milk per 1 g of litter body weight gain

^a,b,c^Means within a row with different superscripts differ using adjusted Tukey method (*P* < 0.05)

### Maternal NR supplementation improved mammary regression and piglet health characteristics

**Fig. 1:**
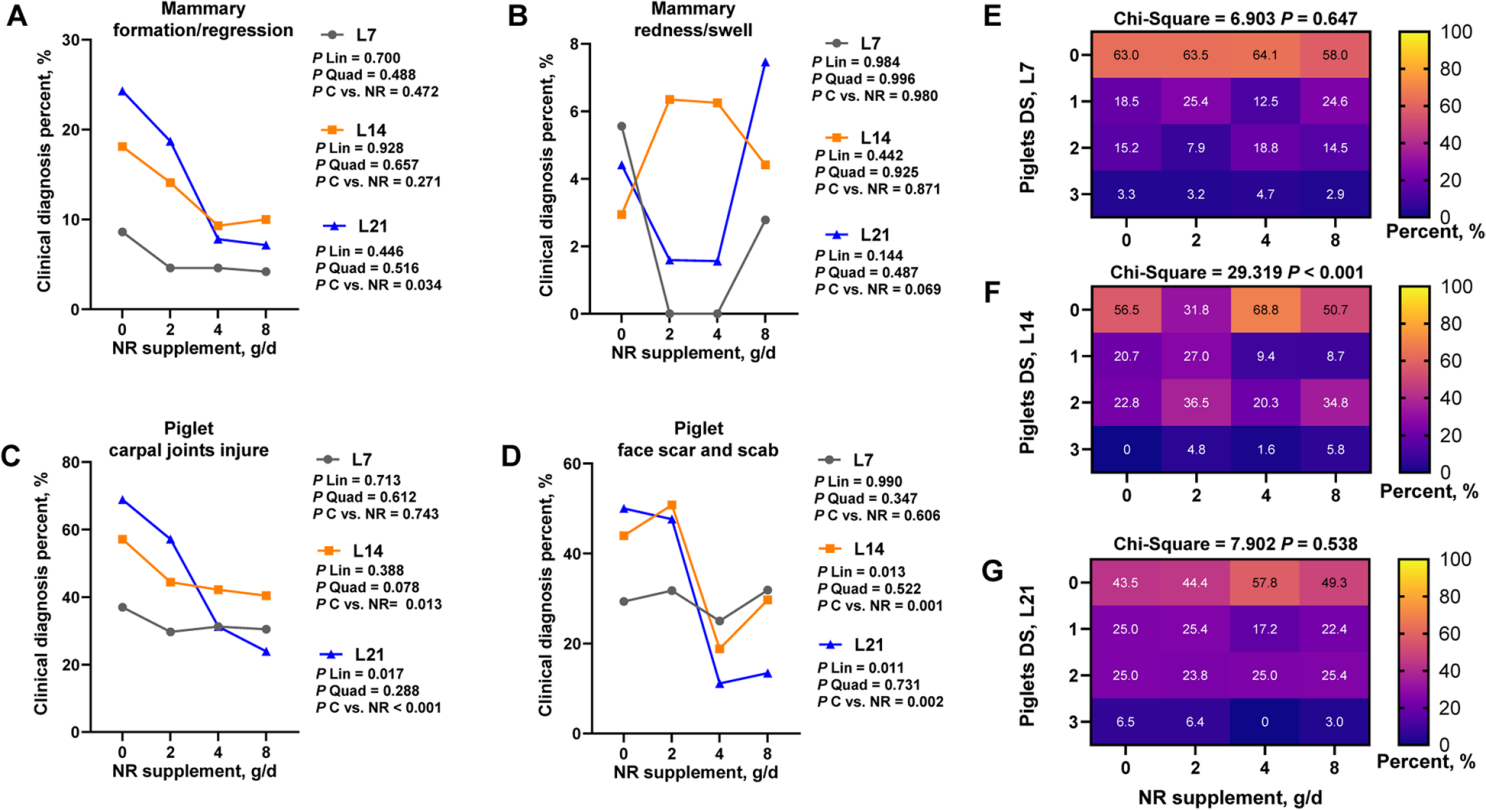
The health score of sow-offspring of late gestation and lactation sows supplemented with increasing levels of nicotinamide riboside. **A** Sows mammary formation/regression. **B** Sows mammary redness/swell. **C** Piglet carpal joints injure. **D** piglet face scar and scab. **E**–**G** piglet diarrhea score at L7, L14, and L21. For **A–** **G** *n* = 65 per group. Data are presented as frequency of diagnosis occurrence (%), C vs. NR means that the comparative analysis of pooled NR supplementation groups (encompassing all dosage gradients) compared with controls. DS: diarrhea score; L7: lactation day 7; L14: lactation day 14; L21: lactation day 21

Mammary gland regression in sows was mildest on lactation day 7 (L7) and most severe on L21 (Fig. 1A). However, 4 or 8 g/d NR maintained consistently low regression incidence (~ 10%), with NR groups exhibiting significantly improved regression rates versus controls on L21 (*P* = 0.034). Mammary redness showed no significant differences (Fig. 1B). Skin injury in piglets from nursing competition followed similar temporal patterns (mildest at L7, severest at L21) (Fig. 1C, D). NR supplementation significantly reduced lesion incidence on lactation day 14 (L14) and L21 with a linearly response to NR (*P* < 0.05) (Fig. 1C, D). For diarrhea scores, no intergroup differences occurred at days 7 or 21, but piglets from sows receiving 4 g/d NR had the highest proportion of score 0 (no diarrhea) on L14, and scores 2–3 were most prevalent in 2 and 8 g/d groups.

### Maternal NR supplementation changed the plasma metabolites on late gestation and lactation

As delineated in Table 5, plasma alkaline phosphatase (ALP) levels were significantly reduced in NR-supplemented sows versus controls on G110 (*P* = 0.003), while alanine aminotransferase (ALT) activity showed a dose-dependent linear decrease (*P* = 0.025). No significant differences were observed in aspartate aminotransferase (AST), glucose (GLU), non-esterified fatty acids (NEFA), triglycerides (TG), total cholesterol (TC), or urea levels, though GLU exhibited numerical elevation in the NR group (*P* = 0.230). During lactation, NR supplementation again significantly reduced ALP (*P* = 0.019) with the minimum observed at 4 g/d, numerically (quadratic, *P* < 0.05). ALT, AST, GLU, and NEFA showed no significant difference compared with controls, but GLU displayed a numerical decrease, opposite to a gestational trend. Notably, TG, TC, and urea were significantly decreased in NR-fed sows with the minimum observed at 4 g/d, numerically (linear and quadratic,* P* < 0.05).

**Table 5:** The plasma metabolites of late gestation and lactation sows supplemented with increasing levels of nicotinamide riboside^1^

Items	NR supplement, g/d				SEM	*P*-values		
	0	2	4	8		Lin	Quad	C vs. NR
On G110								
ALP, mmol/L	72.33^a^	45.68^b^	46.47^b^	53.86^b^	6.51	0.151	0.008	0.003
ALT, U/L	43.51	43.61	36.62	35.92	3.10	0.025	0.752	0.157
AST, U/L	32.35	31.30	32.85	30.44	2.75	0.679	0.787	0.799
GLU, mmol/L	4.04	4.33	4.17	4.42	0.18	0.226	0.917	0.230
NEFA, mmol/L	505.62	448.87	494.63	551.29	45.50	0.294	0.334	0.887
TG, mmol/L	0.15	0.19	0.14	0.17	0.02	0.928	0.754	0.631
TC, mmol/L	1.14	1.14	1.06	1.04	0.06	0.146	0.819	0.347
UREA, mmol/L	2.34	2.25	2.27	2.34	0.17	0.898	0.644	0.787
On L14								
ALP, mmol/L	82.30^b^	62.85^a^	55.39^a^	61.50^a^	8.03	0.112	0.063	0.019
ALT, U/L	47.65	51.04	51.04	49.12	2.79	0.846	0.352	0.387
AST, U/L	27.48	22.62	27.06	25.43	1.91	0.838	0.567	0.234
GLU, mmol/L	5.03	4.77	4.81	4.81	0.18	0.513	0.468	0.121
NEFA, mmol/L	59.81	51.15	67.20	66.57	6.51	0.227	0.909	0.808
TG, mmol/L	0.31^a^	0.21^b^	0.18^b^	0.17^b^	0.03	0.03	0.051	< 0.01
TC, mmol/L	2.17^a^	1.83^b^	1.79^b^	1.65^b^	0.07	< 0.01	0.060	< 0.01
UREA, mmol/L	4.87^a^	3.64^c^	3.99^bc^	4.44^ab^	0.235	0.702	0.010	0.030

*ALP* Alkaline phosphatase, *ALT* Alanine aminotransferase, *AST* Aspartate aminotransferase, *GLU* Glucose, *NEFA* Non-esterified fatty acids, *TG* Triglycerides, *TC* Total cholesterol

^1^Means for each dependent variable represent 13 to 15 observations per treatment after the removal of outliers

^a,b,c^Means within a row with different superscripts differ using adjusted Tukey method (*P* < 0.05)

### Maternal NR supplementation enhanced antioxidant capacity and reduced inflammation state of sows at late gestation and lactation

**Fig. 2:**
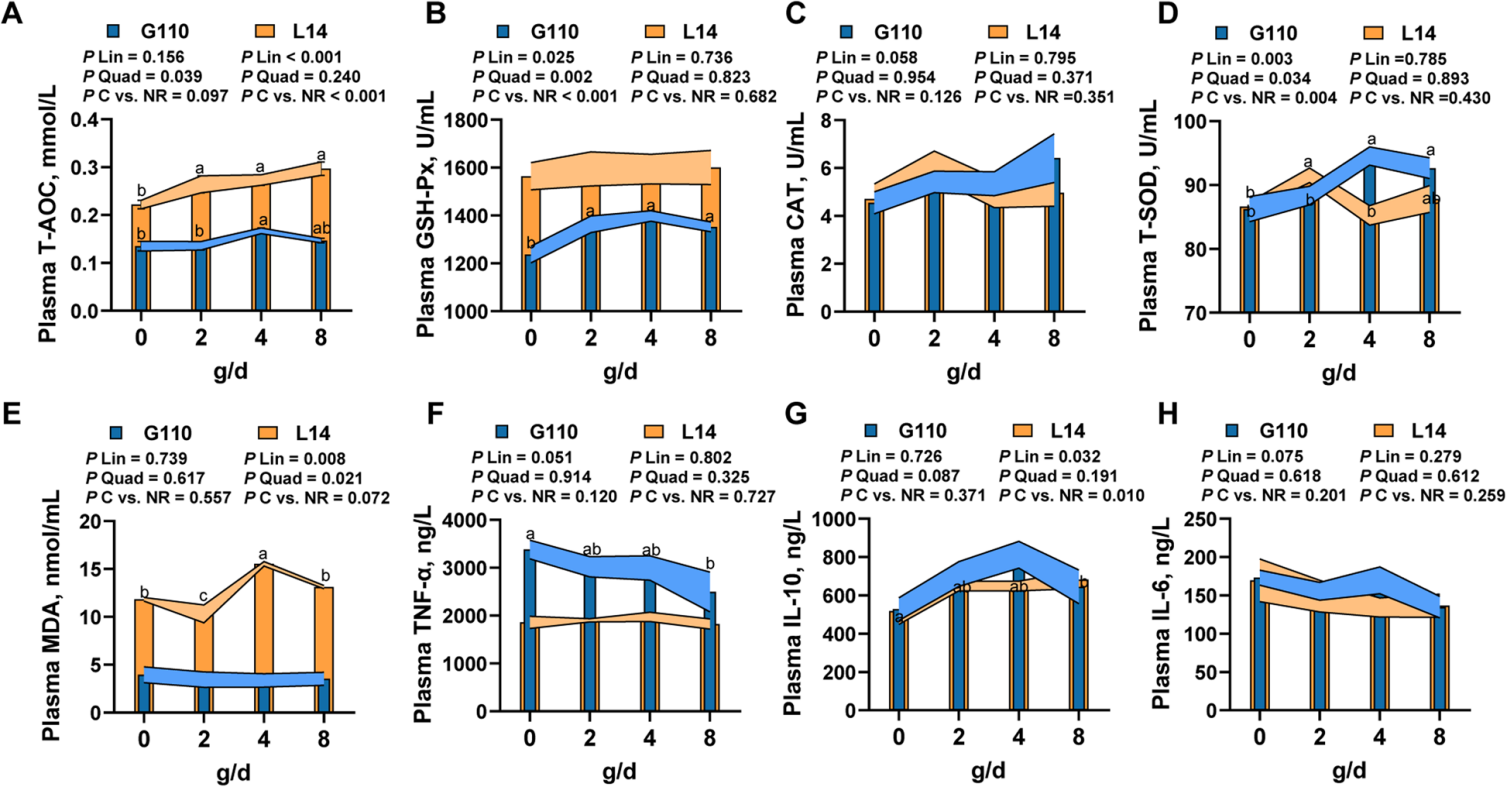
The antioxidant capacity and inflammation state of late gestation and lactation sows supplemented with increasing levels of nicotinamide riboside. **A** Plasma T-AOC. **B** Plasma GSH-Px. **C** Plasma CAT. **D** Plasma T-SOD. **E** Plasma MDA. **F** Plasma TNF-α. **G** Plasma IL-10. **H** Plasma IL-6. For **A**-**H** *n* = 15 per group. Data are presented as mean ± SEM. Orange represents the lactation stage and blue represents the gestation stage, forming orange and blue error bands over the bar plots to better visualize the linear or quadratic effects of different NR supplementation levels. Different letters above error bands of the same color indicate statistical significance using adjusted Tukey method (*P* < 0.05). T-AOC: Total antioxidant capacity; GSH-Px: Glutathione Peroxidase; CAT: Catalase; T-SOD: Total superoxide dismutase; MDA: Malondialdehyde; TNF-α: Tumor necrosis factor-alpha; IL-10: Interleukin-10; IL-6: Interleukin-6

Compared with controls on G110, NR supplementation significantly increased plasma GSH-Px (*P* < 0.001) and T-SOD (*P* = 0.004), with a tendency toward elevated T-AOC (*P* = 0.097). All three antioxidants exhibited quadratic dose responses (*P* < 0.05), peaking at 4 g/d (Fig. 2A–D). During lactation (L14), NR supplementation linearly increased plasma T-AOC (*P* < 0.001), while GSH-Px, CAT, and T-SOD showed no significant differences compared with controls (Fig. 2A–D). Conversely, plasma MDA was significantly elevated in the NR group (linear and quadratic,* P* < 0.05), where the highest MDA was observed at 4 g/d (Fig. 2E). For inflammatory cytokines on G110, NR supplementation had a tendency to linearly suppressed TNF-α (*P* = 0.051) and IL-6 (*P* = 0.075), while IL-10 was quadratically elevated (*P* = 0.087). On L14, only IL-10 increased linearly with NR supplementation (*P* = 0.032) (Fig. 2F, G).

### Maternal NR supplementation changed the milk composition and milk lipid distribution

**Fig. 3:**
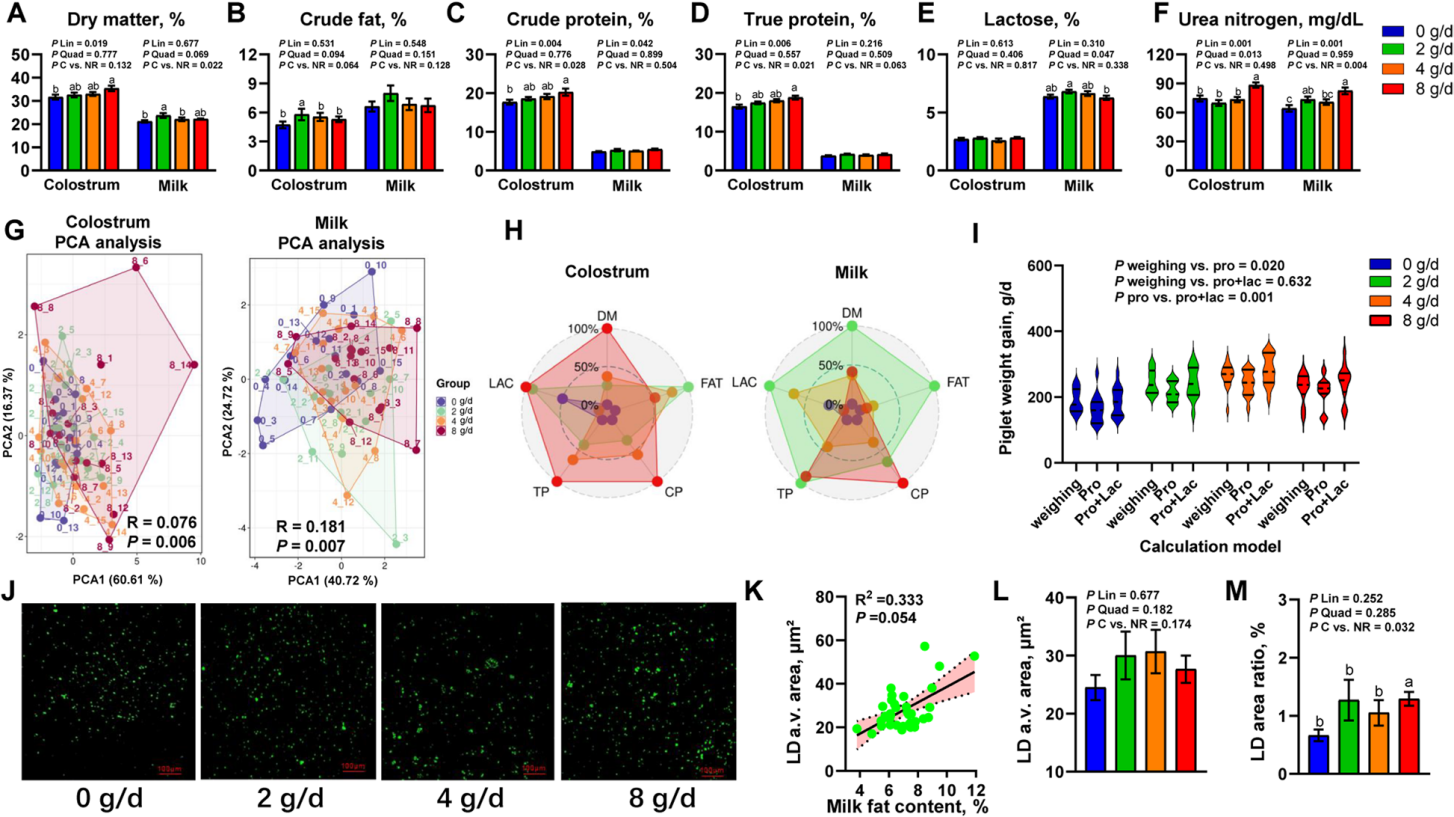
The milk composition and milk lipid distribution of late gestation and lactation sows supplemented with increasing levels of nicotinamide riboside. **A**–**F** Colostrum and milk composition. **G** Principal component analysis (PCA) of colostrum and milk compositional parameters based on ANOSIM analysis. **H** Radar chart of colostrum and milk compositional parameters (Percentage normalization). **I** The piglet weight gain estimation with different models. **J** Representative images of milk Lipid droplets (LD) were stained with BODIPY 493/503 (green), Original magnification, 200 ×. **K** Correlation between LD integrated fluorescence area and milk fat content, shaded area: 95% CI. **L** and **M** Milk LD average area and total area ratio. For **A–M** *n* = 15 per group. Data are presented as mean ± SEM. Different letters in the mean values indicate significant differences using adjusted Tukey method. Pro: Crude protein; Lac: Lactose; a.v.: average

Compared with controls, the dry matter, crude protein, true protein, and urea nitrogen content of colostrum increased linearly in response to NR supplementation, peaking at 8 g/d (*P* < 0.05), while crude fat exhibited a quadratic trend (*P* = 0.094), peaking at 2 g/d (Fig. 3A–F). In mature milk, NR groups showed elevated dry matter (*P* = 0.022), true protein (*P* = 0.063), and urea nitrogen (*P* = 0.004) versus controls, with dry matter and lactose demonstrating quadratic increases (*P* < 0.05) peaking at 2 g/d, while crude protein and urea nitrogen increased linearly (*P* < 0.05) maximized at 8 g/d (Fig. 3A–F). PCA analysis revealed significant separation between control and NR-fed sows for colostrum and mature milk composition (*P* < 0.05) (Fig. 3G). Radar plots indicated controls clustered centrally (lower levels), the 4 g/d group showed intermediate levels, while colostrum in the 8 g/d group and mature milk in the 2 g/d group occupied peripheral regions (superior levels) (Fig. 3H). Furthermore, the model of Hojgaard et al. [36] integrating milk crude protein and lactose accurately estimated piglet daily gain in this study (Fig. 3I). Milk BODIPY staining revealed a positive correlation between lipid droplet mean area and milk fat percentage (R^2^ = 0.333, *P* = 0.054) (Fig. 3J, K) with NR significantly increasing total lipid droplet area proportion (*P* = 0.032) despite unchanged mean area (Fig. 3L, M).

### Maternal NR supplementation changed milk metabolome with enriching in NAD^+^ metabolism

**Fig. 4:**
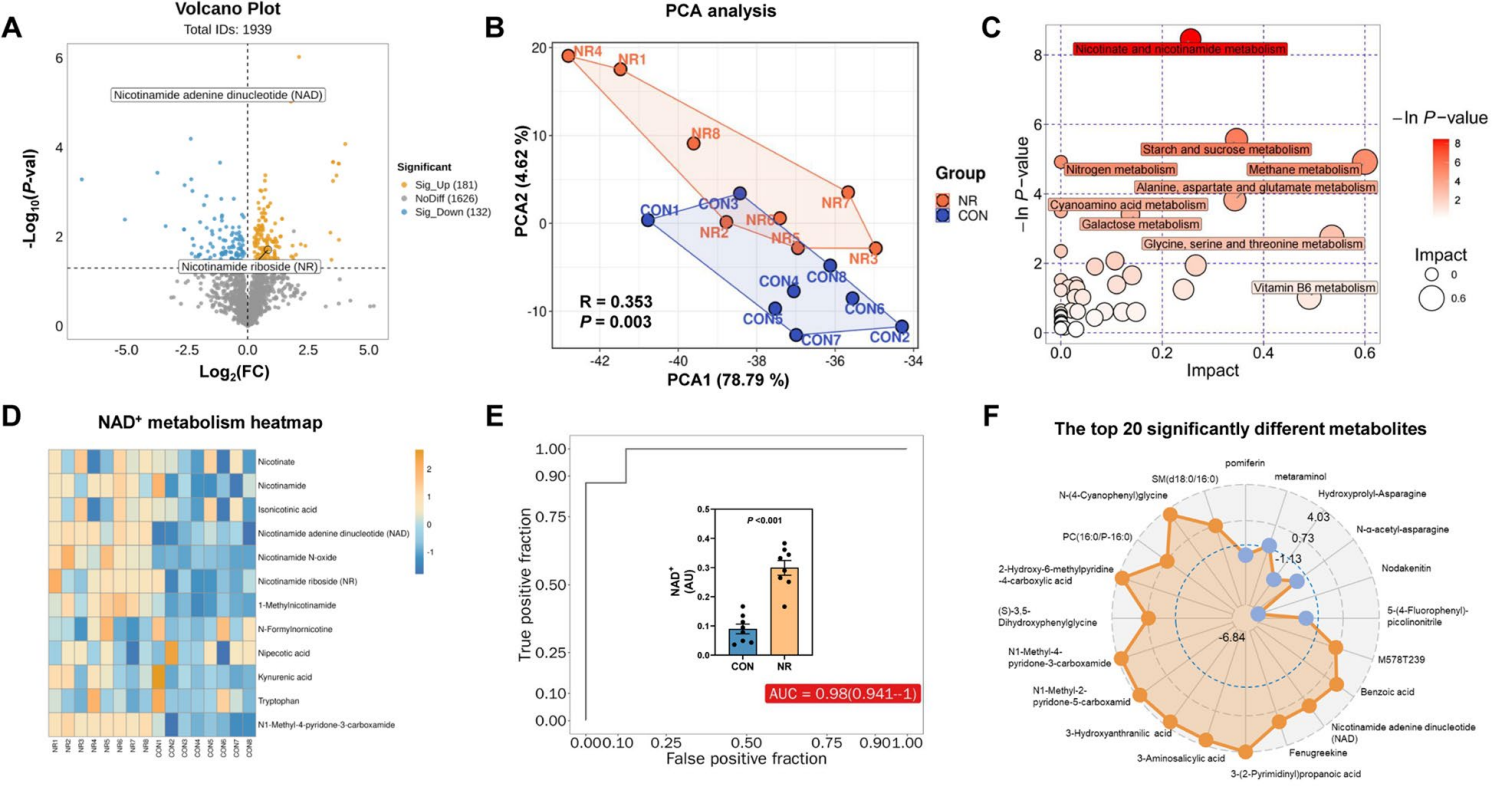
The milk metabolome and nicotinic-nicotinamide pathway of maternal NR supplementation in sows. **A** Volcano plot of 1,939 milk metabolites. Red dots: significantly upregulated in NR vs. CON (*P* < 0.05, FC > 1). Blue dots: downregulated (*P* < 0.05, FC < 1). Dashed lines: Benjamini–Hochberg FDR < 0.05 thresholds. **B** Principal component analysis (PCA) of milk metabolome based on ANOSIM analysis. **C** Metabolic pathway bubble map for the milk metabolome of NR vs. CON. circle area = pathway impact, color = −log_10_(*P*-value). **D** Heatmap representation of metabolites in the nicotinate and nicotinamide metabolism pathway. **E** ROC curve for milk NAD⁺ differential abundance using Student’s *t*-test. **F** The top 20 significantly different metabolites. The substances (Blue dot) within the blue coils represent a decrease (log_2_(FC) < −1). For **A**–**F** *n* = 8 per group. The CON group corresponds to 0 g/d, while the NR group corresponds to 4 g/d. AUC: Area under curve; AU: Arbitrary unit; FC: Fold change

Non-targeted metabolomics of mature milk from control and NR-fed sows (optimal-dose: 4 g/d) identified 1,939 putatively annotated metabolites, with 181 upregulated and 132 downregulated in the NR group (Fig. 4A). PCA analysis revealed clear intergroup separation (*P* < 0.05) (Fig. 4B). In the metabolic enrichment bubble diagram (Fig. 4C), the nicotinamide and nicotinate metabolism pathway was enriched as well as amino acid metabolism, starch/sucrose metabolism, lactose biosynthesis, nitrogen metabolism. As depicted in Fig. 4D, heatmap analysis demonstrated elevated levels of NAD^+^-related metabolites in NR group milk versus controls, including nicotinamide, NAD^+^, nicotinate, NR, Nicotinamide N-oxide, NR, and NAD^+^ terminal metabolites (1-methylnicotinamide and N1-methyl-4-pyridone-3-carboxamide). 4 g/d NR supplementation significantly upregulated 3.34 folds NAD^+^ content versus controls (*P* < 0.001), with ROC analysis confirming its diagnostic potential (AUC = 0.98, 95% CI: 0.94–1.00) (Fig. 4E). Among the top 20 differentially abundant metabolites (Fig. 4F), increased species included NAD⁺ metabolites (NAD⁺, N1-methyl-4-pyridone-3-carboxamide, N1-methyl-2-pyridone-5-carboxamide, nicotinamide, 3-hydroxyanthranilic acid), polar lipids (SM, PC), fenugreekine, and 3-aminosalicylic acid, while pomiferin, metaraminol, nodakenin, and asparagine derivatives decreased.

### Maternal NR supplementation enhanced gut microbiota-derived SCFAs and NAD^+^ metabolism in sows and offspring

**Fig. 5:**
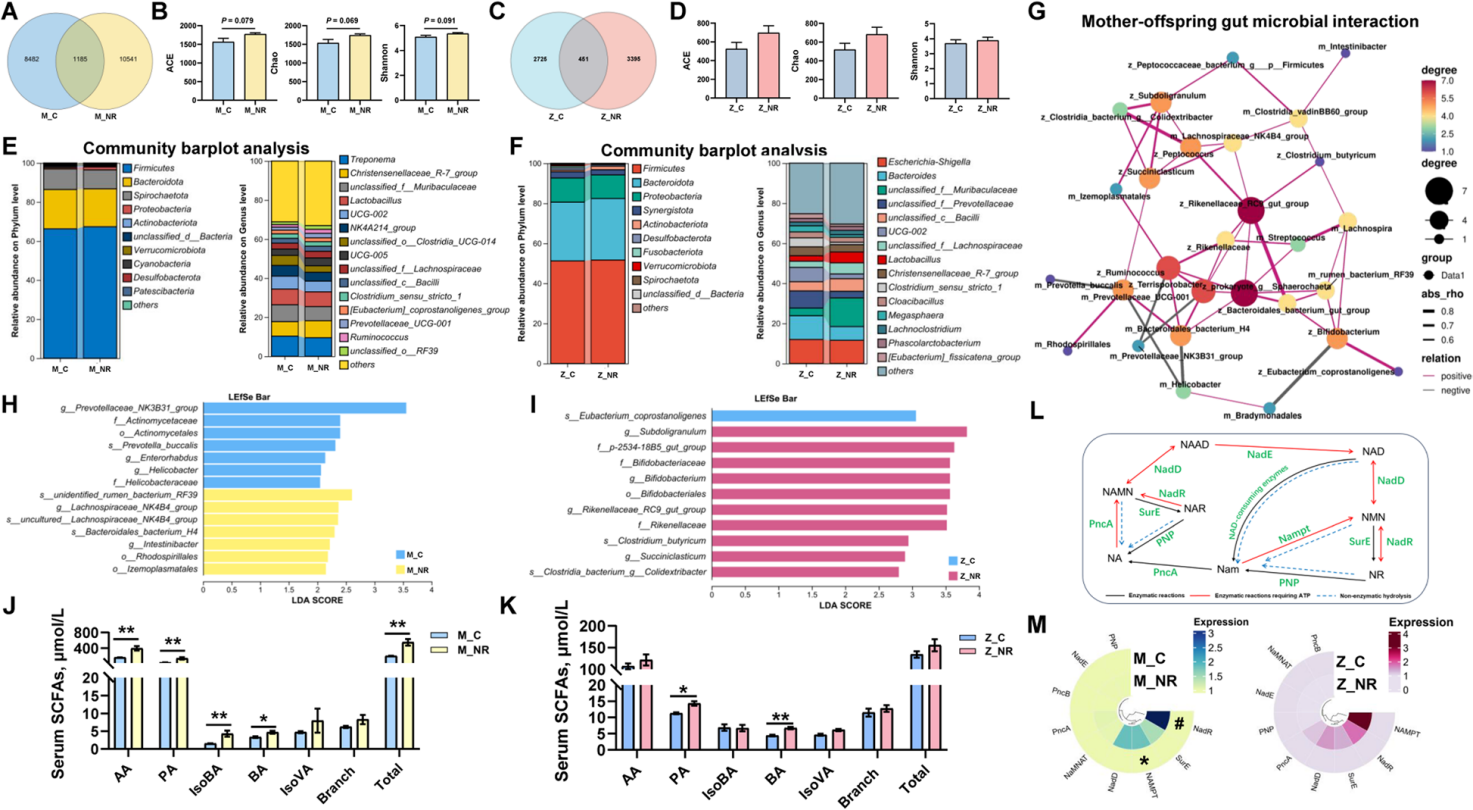
The gut microbiota-derived SCFAs and NAD^+^ metabolism of maternal NR supplementation in sows and offspring. **A** Venn diagrams depicting the number of shared and exclusive ASV in sow microbiota (L14). **B** Sow α-diversity indices (ACE/Simpson/Chao). **C** Venn diagram of piglet ASVs (L14). **D** Piglet α-diversity indices (ACE/Simpson/Chao). **E**, **F** Gut microbial community bar plot at the phylum and genus level of sows and piglet. **G** Microbial interaction network: top 30 differential microbes of sow and piglet; red/gray lines = positive/negative correlations (*P* < 0.05 and |r|> 0.4), width = correlation strength; node size/color = connectivity. **H**, **I** LEfSe analysis of sows and piglets. **J**, **K** Plasma SCFAs concentration of sows and piglets. **L** Schematic representation of reactions involved in the biosynthesis of NAD^+^ in bacteria. **M** PICRUST2 of gut microbiota enzymes related to NAD^+^ metabolism of sows and piglets. For **A**-**F** *n* = 8 per group. Data are presented as mean ± SEM, ^*^ *P* < 0.05, ^**^ *P* < 0.01, ^#^ *P* < 0.1. M_C = Sows received 0 g/d NR; M_NR = Sows received 4 g/d NR; Z_C = Piglets from sows received 0 g/d NR; Z_NR = Piglets from sows received 4 g/d NR; AA = Acetic acid; PA = Propionic acid; IsoBA = Isobutyric acid; BA = Butyric acid; IsoVA = Isovaleric acid; Branch = Branch SCFAs; Total = Total SCFAs

To investigate NR’s impact on sow-offspring gut microbiota, fecal samples from control and NR-fed sows (4 g/d optimal dose) and their piglets were analyzed via 16S rRNA sequencing at lactation day 14. NR sows exhibited higher ASV counts (11,726 vs. 9,667) with trends toward increased α-diversity (ACE/Chao/Shannon indices; *P* < 0.1) (Fig. 5A, B), while their piglets showed elevated ASV counts (3,846 vs. 3,176) but unchanged α-diversity (Fig. 5C, D). Firmicutes, Bacteroidota, Spirochaetota, Proteobacteria, and Synergistota constituted > 96% phylum-level abundance in both sow and piglet, though the top 15 genera differed significantly (Fig. 5E, F). Correlation analysis of the top 30 differential microbes revealed strong sow-piglet microbiota associations (Fig. 5G), with sow-derived *Lachnospiraceae_NK4B4*, *Lachnospira*, *Ruminobacter*, *Prevotellaceae_UCG-001*, *Clostridia_vadinBB60*, and *Bacteroidales_bacterium_H4* identified as core colonizers that shaped piglet microbial community. LEfSe analysis showed controls enriched in *Enterorhabdus*, *Prevotella buccalis*, and *Helicobacteraceae* (sows) and *Eubacterium coprostanoligenes* (piglets), whereas NR groups were enriched in *Ruminococcus*, *Bacteroidales_bacterium_H4*, *Intestinibacter*, and *Rhodospirillales* (sows), along with *Bifidobacterium*, *Subdoligranulum*, *Rikenellaceae_RC9*, *Clostridium butyricum*, and *Succiniclasticum* (piglets) (Fig. 5H, I). Moreover, NR elevated serum SCFAs in sows (acetate/propionate/isobutyrate/butyrate/total SCFAs; *P* < 0.05) and piglets (propionate/butyrate; *P* < 0.05) (Fig. 5J, K). Bacterial NAD⁺ synthesis pathways (Fig. 5L) indicated that NR conversion to NAD⁺ depends on ATP supply via 2–3 enzymatic steps (*NadR*/*NAPMT*-dependent) or via nicotinamide (NAM) conversion, which requires multi-step processes (NAM → NaMN → NaAD⁺ → NAD⁺). PICRUSt-based metagenomic prediction indicated significantly upregulated NAD synthesis genes (*NadR*, *NAPMT*) exclusively in NR sow feces (KEGG ko00760), with numerical increases in piglet feces (Fig. 5M), and *NadR*, *NAMPT*, *SurE*, and *NadD* were dominantly expressed in fecal microbiota genomes of sow and piglet.

## Discussion

The intensified energy demands of fetal growth and mammary lactogenesis in hyper-prolific sows impose substantial metabolic burdens [4, 5]. Although previous studies have described mechanisms by which NAD⁺ replenishment mitigates stress-induced reproductive dysfunction [21–24], research addressing its application to enhance sow reproductive outcomes and regulate metabolic homeostasis remains scarce. Here, our findings revealed that maternal NR supplementation improved sow performance by enhancing within-litter uniformity and increasing milk yield. Specifically, NR dynamically reprogrammed maternal metabolism, and milk metabolomics together with gut microbiota profiles converged on an NAD⁺-centred remodeling that optimized mammary lactogenesis and systemic metabolic efficiency.

Within modern prolific sows, uterine capacity constraints exacerbate intrauterine growth restriction (IUGR) and low birth weight (LBW) due to placental oxidative stress and nutrient competition [4, 5]. In this study, NR increased the 10^th^ percentile litter weight while reducing LBW incidence and weight variability, concurrently attenuating late-gestation fetal mummification, rescuing fetal survival and uniformity [32]. Elevated maternal NAD⁺ demand during pregnancy [20, 21] and in pathological states such as preeclampsia has been reported [25], wherein placental NAD⁺ depletion directly escalates IUGR risk [39]. Previous evidence shows that 200 mg/kg NR restores placental NAD⁺, improves mitochondrial function and reduces inflammation/oxidative stress in rodents [26]. Mechanistically, NAD^+^ governs placental homeostasis via *SIRT1/3*-mediated epigenetic regulation of trophoblast function and vascularization [40, 41] or enhances maternal resilience against redox-inflammatory pathology [13]. In keeping with these mechanisms, NR reduced apoptosis and ROS and increased antioxidant enzymes in other models [42], findings that align with the enhanced gestational antioxidant capacity observed in our sows. Moreover, NR-supplemented sows exhibited numerically heightened late-gestation blood glucose and reduced backfat loss, indicative of altered maternal energy balance that prioritized nutrient partitioning toward fetal/uterine growth, facilitating accelerated fetal mass accretion (35% total gain) in late gestation [43]. Prior work with other NAD⁺ precursors (e.g. nicotinamide) reported delayed glucose clearance in cattle [15, 16], and rodent studies indicate that 500 mg/kg NR rescues fetal-placental growth via enhanced hepatic gluconeogenesis and glycemia [25]. Such prenatal metabolic adjustments likely contributed to the shorter farrowing duration and reduced birth intervals observed in NR groups, and to lower postpartum body temperatures, cumulatively diminishing oxidative stress and inflammation [2]. Although links between NAD⁺ and parturition physiology merit further study, interactions among bioactive prostaglandins, oxytocin and NAD⁺ regulators in hypothalamic–pituitary circuits are plausible mediators [44, 45]. Thus, the NR dosage here aligns with clinical/FDA guidelines, corresponding to efficacious 200–800 mg/kg BW rodent equivalents, confirming dose-responsive efficacy in embryonic development and parturition. This suggests that the potential of NAD⁺ during the reproductive process [20, 21], particularly in high-energy-demanding developmental stages including parity, breed, and high-prolific levels has yet to be fully explored.

Maternal metabolic status during gestation strongly influences subsequent lactation [1–3]. Gestation-to-lactation transitions are associated with increased mammary and systemic NAD⁺ demand [21, 46, 47]. Our prior research showed that oral NR improved mammary NAD⁺ biosynthesis and drove alveolar proliferation and lactogenic capacity [21]. Similarly, 750 mg/kg NR elevated tissue NAD⁺ and milk yield, with augmented prolactin signaling in rat models [48]. In line with those findings, we found that NR (optimally 4 g/d NR) significantly improved weaning survival, litter parameters, and milk yield while mitigating mammary involution and piglet competition injuries. NR-fed sows had higher lactational total feed intake but reduced triglycerides and total cholesterol, with numerically lower plasma glucose, suggesting enhanced appetite and more efficient nutrient uptake by the mammary gland [3, 49]. The efficiency optimization was superior to mobilizing body reserves compensating for lactation [50], which reduces excessive body loss that can lead to culling or failure to rebreed. Although high doses of NR (8 g/d) exhibited certain improving effects on lactation performance, its inherent bitter taste can compromise palatability and reduce feed intake, particularly during the first week postpartum. Attention must be paid to backfat loss so as to avoid reproductive impairments. Despite prolong lactation duration and higher milk output can increase oxidative burden [51], we observed a dose-dependent rise in plasma MDA, a lipid peroxidation biomarker, that peaked at 4 g/d NR accompanied by increased plasma T-AOC, suggesting that NR enhanced antioxidant defenses and tried to maintain redox balance during elevated lactational demand. Furthermore, milk analyses revealed elevated developmentally critical components in colostrum and mature milk from NR-fed sows, with the model of Hojgaard et al. [36] substantiating that NR-enhanced milk quality drives neonatal growth. Milk lipid droplet area/composition peaked at 2 or 8 g/d, explaining L14 diarrhea via nutrient-excess-driven digestive stress [52]; by contrast, 4 g/d NR appeared to provide the best balance of yield and nutrient quality for optimal lactation. Interestingly, milk metabolomics at this dosage identified NAD⁺-metabolome enrichment with increased NAD⁺ precursors (nicotinamide/nicotinamide N-oxide/NR) and terminal metabolites (1-methylnicotinamide and N1-Methyl-4-pyridone-3-carboxamide). We also detected higher fenugreekine and altered aspartate levels in milk from NR-treated sows. Fenugreekine supports NAD⁺ synthesis and has antioxidant properties that may ameliorate insufficient lactation [53, 54], and prior blood metabolomic studies reported NR-associated increases in fenugreekine [55]. The reduction in aspartate could reflect its utilization as a substrate for NAD⁺ biosynthesis via L-aspartate oxidase [56]. Collectively, these changes indicated amplified mammary NAD⁺ flux and increased NAD⁺ content in milk [21, 48], which can directly support offspring development [48, 57]. It has been established that milk contains NR that can be absorbed by offspring. Subsequent research is required to accurately assess the neonates' actual intake and its long-term efficacy. Additional milk metabolites (e.g., phosphatidylcholine, 3,5-dihydroxyphenylglycine, 3-aminosalicylic acid) identified here likely contribute to neonatal intestinal barrier function and to microbial SCFA synthesis, further promoting offspring growth [58, 59]. Therefore, NR supplementation optimized milk quality, as well as NAD⁺ metabolites and bioactives, driving offspring growth during lactation.

Gut microbiota critically modulates mammalian reproductive physiology [27], and further maintains host NAD⁺ turnover [19, 28]. Maternal NR reshaped sow and piglet microbiota in a manner consistent with enhanced NAD⁺ conversion and metabolic reprogramming. Mirroring murine reports that NR increases α-diversity [55], we observed elevated maternal microbial diversity and synchronous enrichment of beneficial taxa across sow–offspring pairs, changes that are important for progeny growth and resilience. NR reduced potentially pathogenic genera (*Enterorhabdus*, *Prevotella buccalis*, *Helicobacteraceae*, *Eubacterium coprostanoligenes*), while enriching beneficial taxa (*Ruminococcus*, *Lachnospiraceae*, *Bacteroidales H4*, *Rhodospirillales*, *Bifidobacterium*, *Rikenellaceae_RC9*) [60, 61]. Certain taxa (*Bacteroidales H4* and *Bifidobacterium*) appear to thrive in the presence of NAD⁺ precursors [62], and in vitro studies have shown NR-driven expansion of *Ruminococcus* and *Lachnospiraceae* [55]. The involvement of microbes in NAD⁺ synthesis through enzymatic expression is complex, which is essential for NAD⁺ precursors supplementation to exert therapeutic functions [55]. Genomic inference further indicated upregulation of microbial NAD⁺ salvage enzymes (*NadR*/*NAMPT*) following NR supplementation, supporting efficient gut NR → NAD⁺ conversion and a microbial contribution to host-usable NAD⁺ pools [19, 28], supporting milk NAD⁺ enrichment. Concurrent pathogen reduction likely limited gut inflammation-driven NAD⁺ depletion (e.g., via macrophage *CD38* activation) [63], thereby enhancing host NAD⁺ homeostasis. Gut microbiota modulates host NAD⁺ bioavailability and also govern reciprocal host-microbe interactions mediated by bacterial metabolites. NR additionally enriched SCFA-producers (*Lachnospiraceae*, *Ruminococcus*, *Intestinibacter*, *Subdoligranulum*, *Clostridium butyricum*, *Succiniclasticum*) in sows and piglets, corroborating reports of NR-upregulated SCFA-producing genera and KEGG orthology genes for butyrate synthesis [55, 64]. In this study, NR supplementation elevated SCFAs fermentation into host circulation, employing energy metabolism for milk synthesis [65] and antioxidative effects [66], wherein elevated butyrate in piglets may provide antimicrobial, anti-inflammatory and antioxidative benefits that contribute to improved intestinal growth [67]. Thus, maternal NR supplementation appears to orchestrate a coordinated microbiota restructuring that both activates microbial NAD⁺ biosynthesis and augments SCFA production, collectively sustaining host NAD⁺ utilization and metabolic adaptation to meet the high energetic demands of reproduction and lactation.

## Conclusions

Hyper-prolific sows encounter significant physiological stressors that compromise pregnancy outcomes and lactation performance. Maternal NR supplementation from late gestation to lactation enhanced sow performance by improving litter weight uniformity and lactation performance, concomitant with optimized metabolic homeostasis. The response was mediated by coordinated milk-gut metabolic remodeling that amplified NAD⁺ biogenesis and SCFA production to sustain host NAD⁺ utilization and metabolic adaptation. These findings propose maternal 4 g/d NR intervention as a novel strategy to enhance mammary lactogenesis and metabolic efficiency in modern sow production.

## Data Availability

All data necessary to support the conclusions of this study is either included in the paper. The datasets generated and/or analyzed during the current study are available upon reasonable request from the corresponding author.

## Abbreviations

ADFI: Average daily food intake
ADG: Average daily weight gain
BCS: Body condition score
BF: Backfat depth
BW: Body weight
CRL: Crown-rump length
CV_BW_: Coefficient of variation of birth weight
DS: Piglet diarrhea score
IUGR: Intrauterine growth retardation
LBW: Low birth weight
LD: Lipid droplets
LDA: Linear discriminant analysis
NAD⁺: Nicotinamide adenine dinucleotide
NAMPT: Nicotinamide phosphoribosyl transferase
NadR: NA/NAM riboside kinase
NA: Niacin
NAM: Nicotinamide
NR: Nicotinamide riboside
RT: Rectal temperature
SCFAs: Short-chain fatty acids
TFI: Total food intake
